# Profiling of the Polyphenol Content of Honey from Different Geographical Origins in the United States

**DOI:** 10.3390/molecules28135011

**Published:** 2023-06-27

**Authors:** Kate Nyarko, Kaitlyn Boozer, C. Michael Greenlief

**Affiliations:** Department of Chemistry, University of Missouri, 601 S. College Avenue, Columbia, MO 65211, USA; knzhb@mail.missouri.edu (K.N.); kboozer@mail.missouri.edu (K.B.)

**Keywords:** honey, phenolic compounds, total phenolic content, HPLC-MS

## Abstract

The presence of phenolic compounds in honey can serve as potential authenticity markers for honey’s botanical or geographical origins. The composition and properties of honey can vary greatly depending on the floral and geographical origins. This study focuses on identifying the specific markers that can distinguish honey based on their geographical areas in the United States. The main approach presented in this study to identify the geographic origins of honey involves chemometric methods combined with phenolic compound fingerprinting. Sample clean-up and phenolic compound extraction was carried out using solid phase extraction (SPE). Reversed phase liquid chromatography in combination with tandem mass spectrometry were utilized for the separation of the compounds. The honey physicochemical qualities were predominantly determined via spectrophotometric methods. Multivariate statistical tools such as principal component analysis (PCA), analysis of variance (ANOVA), and partial-least squares discriminant analysis (PLS-DA) were employed as both classification and feature selection tools. Overall, the present study was able to identify the presence of 12 potential markers to differentiate the honey’s geographical origins. The total phenolic content ranged from 81.6 to 105.7 mg GAE/100 g corresponding to honey from Colorado and Washington, respectively (GAE: gallic acid equivalents). The regression analysis shows a tendency for the total phenolic content of honey to increase as the color of honey increases. The most important result obtained in this study is the demonstration that the geographical origin of honey plays a critical role in predicting the physical properties and phenolic composition of honey.

## 1. Introduction

Honey is composed primarily of glucose, fructose, and phenolic compounds with lower levels of vitamins, minerals, and proteins. Phenolic compounds are secondary metabolites of plants that are transferred by bees to honey [1]. Honey serves as a good source of bioactive compounds including phenolic acids and flavonoids which contribute greatly to honey’s health benefits due to their antioxidant and anti-inflammatory properties [2,3]. The use of phenolic compounds as potential authenticity markers has been proposed in honey research studies [4,5,6,7]. The composition of honey is highly diverse due to the differences in its floral or geographical origins. Other factors such as environmental, seasonal, and processing conditions of honey can influence its composition [8,9,10]. Honey can be classified as either monofloral or multifloral depending on whether it is derived from a single botanical source or multiple sources, respectively [11]. Botanical origin is typically known to influence the commercial value of honey, especially for monofloral types. Regardless of the botanical origin, there are several factors, including the geographical origin, that impact honey quality and market value. The need to understand the natural, cultural, and social factors of various geographical origins and their influence on the composition and sensory characteristics of honey has generated considerable interest to investigate honey in recent years. Moreover, the emerging trend of identifying important markers for honey’s geographical origins has made it possible to detect adulterants in honey.

The traditional liquid–liquid extraction technique is by far the most used method for the extraction of phenolic compounds in honey as a sample preparation method. This technique often requires large sample and solvent volumes, while low extraction efficiencies have been reported [12,13]. Solid phase extraction (SPE), on the other hand, yields superior extraction efficiency with minimal solvent volumes [14,15]. The determination of honey’s quality characteristics including color, moisture content, pH, free acidity, and sugars have become routine in honey discriminatory studies. This is mainly due to the simple sample analysis required. The screening of phenolic compounds in honey is predominantly executed through the use of chromatographic separation coupled with tandem mass spectrometry. Reversed-phase liquid chromatography was used here to ensure effective separation of the phenolic compounds. Common phenolic compounds in honey reported by various authors include 4-hydroxybenzoic acid, p-coumaric acid, vanillic acid, caffeic acid, syringic acid, gallic acid, quercetin, kaempferol, myricetin, pinobanksin, pinocembrin, and chrysin [15,16,17,18,19]. While the phenolic compounds present in the honey may differ based on geographical or floral origin, it is also dependent on its physicochemical properties such as color, moisture content, and total phenolic content (TPC). Several authors have argued for a possible correlation between the total phenolic content and physicochemical qualities of honey [20,21,22,23]. As an example of this, the color of honey was reported to have a positive correlation with the total phenolic content [24,25]. Thus, the determination of the total phenolic content is crucial in understanding the properties of honey.

The exploration of characteristics markers related to the botanical and geographical origins for honey authentication has been extensively investigated in the literature. However, a major challenge arises from the highly complex and diverse composition of honey [1,12]. Moreover, the content and nature of markers in honey is unknown [26]. The lack of uniformity of the analytical and extraction methods makes the analysis challenging due to the diversification of honeys based on their origins. It is worthwhile mentioning that a great deal of research has focused on honey traceability studies in most parts of the European and Asian countries. However, to date, there is no available literature that compares the phenolic profiles of honeys from various regions in the United States. This is the first study to examine and compare honeys from multiple regions in the United States simultaneously. Hence this study will outline the analytical and chemometric methods for the phenolic compound fingerprinting of honeys from five different geographical origins within the United States. The present study will aim at evaluating the relationship between the total phenolic content and the physicochemical properties of honey.

## 2. Results and Discussion

### 2.1. Physicochemical Properties of Honey

#### 2.1.1. TPC of Honey Compared to Other Geographical Sources

**Figure 1:**
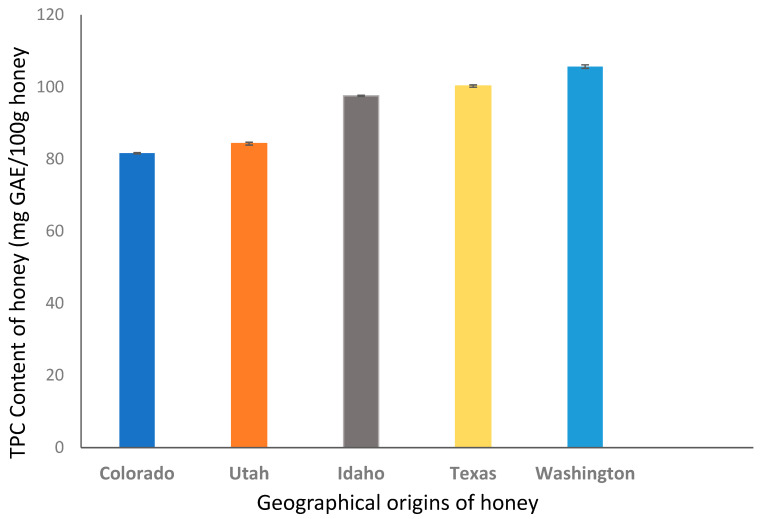
Total polyphenol content (TPC) of honeys from Utah, Idaho, Texas, Washington, and Colorado.

The determination of the total phenolic content is of importance to help trace the floral and geographical origins of honeys [27]. In many instances, the content of phenolic compounds is used to assess the sensory and functional properties of honeys [28]. Although TPC determination has been widely used in estimating the content of phenolic compounds in many food substances, it is not sensitive enough to provide an accurate determination of the total polyphenolic content due to interferences from other compounds present in the complex honey matrix [5]. The total phenolic content was determined in the 15 honey samples analyzed in this study. The average TPC values of the analyzed samples are reported in Table 1. The mean total phenolic concentration ranged between 74.7 and 105.6 mg GAE/100 g, with the highest TPC value corresponding to honey from Washington. The lowest phenolic content was associated with honey from Colorado. Moreover, the ANOVA results showed that the total phenolic content differed significantly between the analyzed honey of different origins (*p* < 0.05). The order of the decreasing total phenolic content (mean values) is as follows: Washington > Texas > Idaho > Utah > Colorado and is shown in Figure 1.

A similar study by Habryka et al. [29] measured TPC and found the values to range from 30.8 to 178.3 mg GAE/100 g in multifloral honeys from Poland. The mean content of phenolic compounds was found as 36.6 ± 1.78 mg GAE/100 g in polyfloral honeys from northern Belgium [12]. Spanish honeys analyzed by Martin and co-workers [30] were found to have an average TPC value of 102 mg GAE/100 g which is similar to the TPC value obtained for Texas honeys. In a similar study, the highest average TPC of Brazilian honeys examined by Nascimento et al. [31] was determined to be 100 mg GAE/100 g, which is in concordance with those previously reported in honeys from Texas and Idaho. The total phenolic content of Washington honeys is similar to TPC amounts for Turkish and Tunisian honeys. In contrast, Bertoncelj et al. [21,32] reported a high concentration of total phenolic compounds for Slovenian honeys. Their measured values were twice the highest TPC content determined in this study. In another study in Cuba, polyfloral honeys [11] were characterized by a lower overall TPC with a highest average value of 54.3 mg GAE/100 g. Overall, our present findings are comparable with previously published data on European honeys from different geographical regions.

The TPC results are also in excellent agreement with a previous work carried out by Zhao et al. [33] who investigated the phenolic compound fingerprints of different monofloral honeys from China. In this study, the authors reported that bright-colored honeys have lower phenolic contents compared with dark colored ones. Our present study also shows the presence of high amounts of polyphenol compounds in colored honeys and vice versa. Dark honeys from Washington and Texas yielded higher phenolic contents of 105.6 and 100.2 mg GAE/100 g, respectively. Conversely, honey samples with lighter color yielded lower total phenolic content as observed for the Colorado honey (81.6 mg GAE/100 g).

#### 2.1.2. TPC vs. Honey Physicochemical Properties

In this study, the physicochemical properties of the samples were analyzed. A given property was then examined to see if there was a relationship with TPC based on their geographical origins. All the analyzed honeys had distinct physicochemical properties. Other than the geographical source, the variation of phenolic content can be attributed to processing factors, climatic conditions of the honey origin, and the botanical source of the honey [5]. Table 1 summarizes the physicochemical properties of the honey samples.

#### 2.1.3. pH of Honey

The pH of honey is an important parameter that can confirm honey deterioration caused by enzymatic activities [34,35]. The determination of pH is equally important to understand the chemical composition of honey that complements its sensory and nutritional qualities [36]. The pH of the honey solutions was measured and the pH ranged between 5.4 and 5.5 as shown in Table 1. The pH values were within the recommended pH range of the Codex Alimentarius Standard Commission [25]. There is a weak to no correlation (r = 0.169) between pH and the total phenolic content of the honey samples. These pH values are comparable with similar studies where the pH levels of honey have been reported [37].

#### 2.1.4. Color of Honey

Although honey color does not greatly affect the quality and price of honey in the United States, it is known to contribute significantly to the sensory and bioactive properties of honey [9,38,39]. The honey solutions were measured spectrophotometrically using the Pfund method as described by Beretta et al. [40]. The color of all the honey samples ranged widely between light amber and dark amber tones [41]. As illustrated in Table 1, we found a significant correlation (r = 0.931) between the color of honey and TPC and conclude that the phenolic composition of honey is characterized by its color and sensory properties. It has been reported elsewhere that Maillard reaction products produced during honey processing and storage can contribute to the formation of honey color [42].

Several studies have linked the color of honey to the presence of flavonoids and phenolic compounds [43]. A study by Ciappini et al. [44] reported a strong positive correlation (r = 0.931) in dark colored multifloral honey from Argentina. A similar study by Al-Farsi et al. [24] found higher TPC amounts in colored Oman honeys (r = 0.974). In comparison, Balcázar-Cruz et al. [45] also found a good linear correlation (r = 0.864) for dark-amber honeys harvested in Mexico. In this study, we also reported a similar trend of increasing TPC as the honey color becomes darker.

#### 2.1.5. Moisture Content of Honey

According to the Codex Alimentarius Standard Commission of honey, the moisture content should not exceed 20% regardless of its botanical or geographical source [37]. Higher moisture content may result in a lower shelf-life or possible honey fermentation [46,47]. Thus, a lower moisture content is recommended to maintain honey quality. In this study, we measured low moisture levels in all the honey samples (Table 1). The reduced moisture content verifies that the shelf-life of the honeys is appropriate. We found weak to no correlation between the moisture level and the TPC content (r = 0.036, *p* < 0.05). The amount of moisture in honey has also been linked to its thermal processing. For instance, it was reported that honey processed at 60 °C had a higher moisture content of 17.98% while relatively lower moisture levels of 16% and 17% were reported at 70 °C and 80 °C, respectively [46]. Moreover, the application of excessive heating temperatures above 80 °C could lead to the formation of undesirable degradation products such as HMF [48].

#### 2.1.6. HMF Determination in Honey

The measured HMF contents of the honey samples are summarized in Table 1. HMF is a widely recognized parameter used to assess honey freshness and quality. Higher HMF concentrations can indicate inappropriate honey storage and/or excessive thermal treatments. A good example was illustrated in a study by Bath et al. [49] that reported a considerable increase in HMF concentration in honey stored over prolonged periods. A similar situation was observed when honey was stored in metallic storage containers [50]. Khalil et al. [51] investigated the HMF concentrations of five Malaysian honey samples stored over a six-month period by HPLC and found the HMF amounts to range from 2.8 to 24.8 mg/kg over the time course. In this study, the HMF content of the honey samples stored over a duration of six months was found to vary between 5.5 and 12.1 mg/kg, which was half the average HMF reported for Malaysian honey. Moreover, the HMF values obtained in this study are well below the permitted range of 40 mg/kg proposed under the Codex Alimentarius Standard Committee (2001) [37], and 80 mg/kg for honeys produced in tropical regions. The effects of HMF concentration on TPC were nearly imperceptible based on the poor correlation value (r = 0.467) obtained. Despite the poor correlation reported in this study, it is important to note that prolonged honey storage may contribute to high HMF levels as evidenced in the previous published studies, but this does not necessarily yield a relationship with its geographical source.

### 2.2. Identification of Phenolic Compounds in Honey

SPE-HPLC/MS was used to examine the presence of polyphenolic compounds in all 15 honey samples from the studied geographical areas. The identification of compounds in this study was based on the comparison of the mass spectra, retention times, and fragmentation patterns of the individual compounds with available standards or by direct comparison with literature data found in mass spectral libraries. Table S1 is a compilation of the individual phenolic acids and flavonoids identified in each analyzed sample, along with their masses, retention times, and major fragment ion masses.

**Figure 2:**
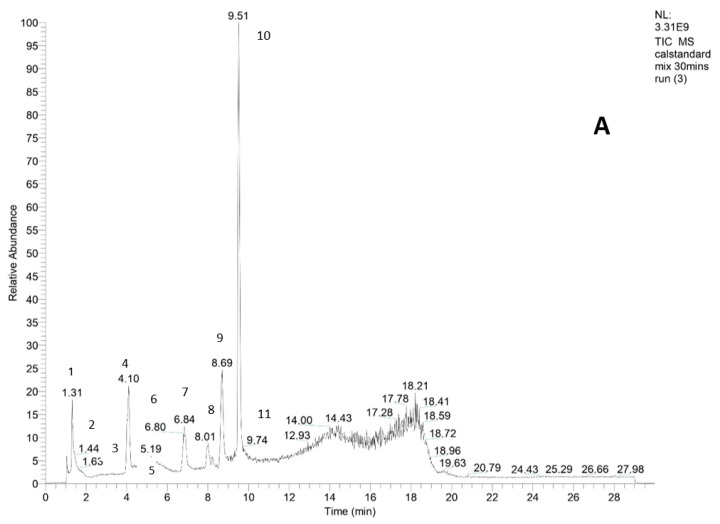

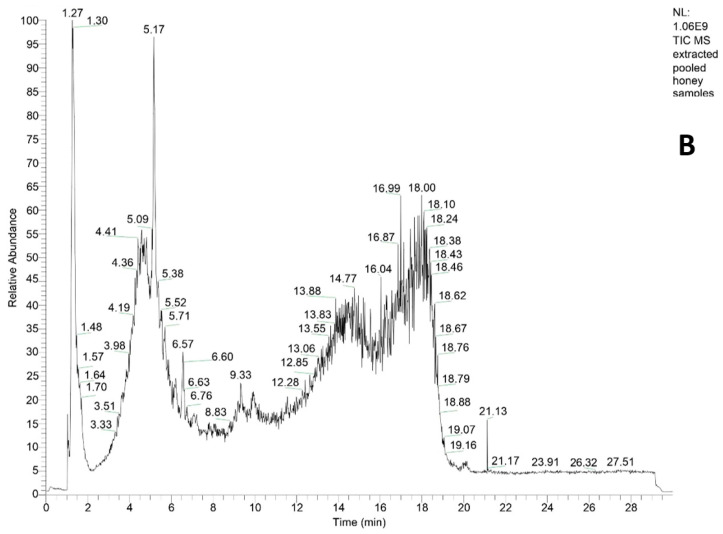
Identifications based on a comparison of LC chromatograms of (**A**) phenolic compound standards and (**B**) a representative extracted honey solution. Peaks’ labels in (**A**) correspond to: sinapic acid (1), caffeic acid (2), vanillic acid (3), gallic acid (4), gentistic acid (5), chlorogenic acid (6), naringenin (7), quercetin (8), kaempferol (9), apigenin (10), rifampicin (11).

Based on our results, we identified a total of 18 phenolic compounds. The compounds are flavonoids (*n* = 13) (quercetin-3-O-(6″-malonyl-glucoside), isorhamnetin, quercetin, kaempferol, naringenin, chrysin, apigenin, pinocembrin, 6-phrenylnarigenin, hesperidin, kaempferol 3-O-rhamnoside, hesperidin, and (+)-catechin 3-O-glucose) and five compounds are phenolic acids (sinapic acid, caffeic acid, 5′5-dihydroferulic acid, ferulic acid, and subaphyllin). Among the phenolic acids, caffeic acid and ferulic acid have been found to demonstrate higher antibacterial activity against several bacterial species [52]. The phenolic compounds with [M-H]^−^ ions at *m*/*z* 223.04, 153.13, 285.19, 271.13, and 269.22 Da for sinapic acid, caffeic acid, kaempferol, naringenin, and apigenin, respectively, were tentatively identified by matching their retention times with that of the available standards presented in Figure 2. The identity of the other compounds was confirmed from their tandem MS/MS mass spectra in combination with the literature data to obtain further information on the structural elucidation of the aglycone derivatives.

Yao et al. [7] found significant amounts of ferulic acid in Australian Eucalyptus honeys. The presence of ferulic acid was also observed in multifloral European honeys. In this study, ferulic acid was detected in honey from Utah. The largest number of compounds were detected in samples from Washington and Colorado. Of the compounds identified, only five were detected simultaneously in all the analyzed samples. These included sinapic acid, caffeic acid, 5,5-diferulic acid, kaempferol, and quercetin-3-O-(6″-malonyl glucoside). Previous studies have also shown the presence of caffeic acid in Polish [53] and European honey varieties [6]. Kaempferol, one of the leading flavonoids in honey and natural foods, was detected in all the honey samples and has been reported earlier by several authors [22,54,55,56]. In the study of Croatian honeys by Kenjeric et al. [56], the presence of kaempferol was detected while Baltrusaityte et al. [54] identified relatively high amounts of kaempferol in Lithuanian honeys.

The phenolic compound profile differed by the presence of some specific compounds in the samples. For example, we detected the presence of flavonoids such as pinocembrin and naringenin in honey samples from Washington and Colorado. Furthermore, the phenolic acid and flavanone derivatives such as subaphyllin and 6-prenylnaringenin, respectively, are rarely reported in honey, yet were identified in samples collected from Utah. On the other hand, the flavonol myricetin was uniquely identified in Texas honey. In the case of Idaho, its polyphenolic chemical profile was marked by the presence of a flavonoid glycoside such as hesperidin. In addition, samples from Washington were characterized by the presence of some common flavonoids such as quercetin, apigenin, naringenin, and isorhamnetin. This is similar to those identified compounds in honeys of different botanical and geographical sources [15,16,29,57,58,59]. The presence of eight phenolic compounds such as 3,4-dihydroxybenzoic acid, 4-hydroxybenzoic acid, caffeic acid, gallic acid, p-coumaric acid, pinobanksin, rutin, and 3-hydroxytyrosol in Spanish-made honey was measured by Garcia et al. [60]. Contrary to the present results, phenolic compounds such as gallic acid, p-coumaric acid, 4-hydroxybenzoic acid, rutin, and luteolin that are well documented in the literature for both monofloral and multifloral honeys were not present in any of the samples. The varying honey phenolic profiles can be linked to the floral and sensory properties of honey and the climatic and environmental conditions of the production regions [5].

### 2.3. Chemometric Tools for Honey Classification Analysis

**Figure 3:**
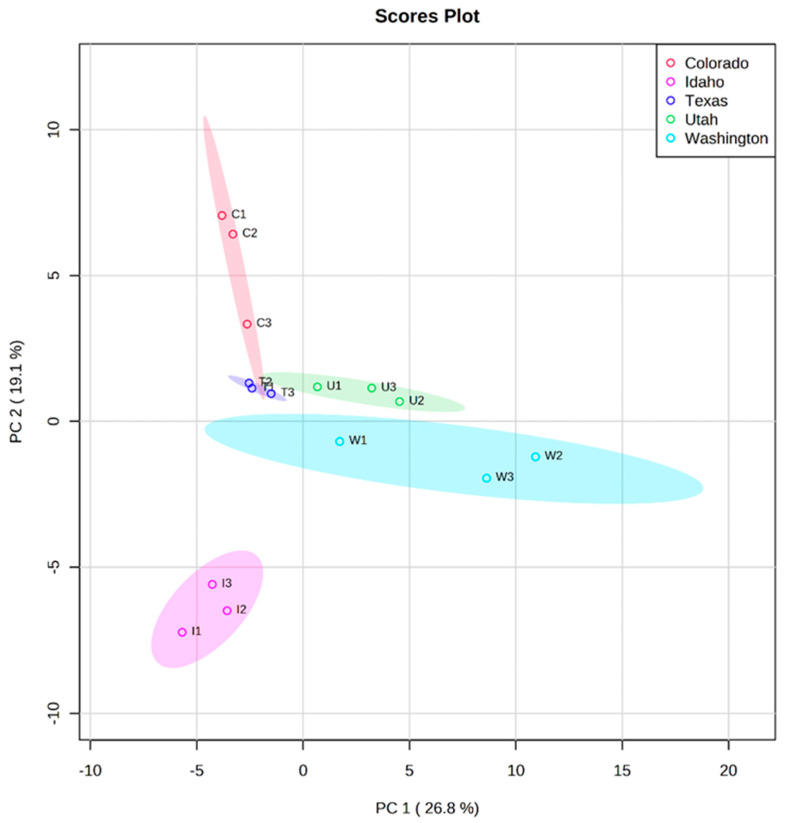
Principal component analysis (PCA) for discrimination of honey geographical sources.

The honey samples were subjected to ANOVA to determine features of statistical significance. Of the compounds, 18 out of the 30 were found to be statistically significant (*p* < 0.05) for the geographical discrimination of the investigated honeys which were selected and later submitted for PCA analysis. Multivariate statistical tools such as PCA, and supervised PLS-DA were used to classify the honeys based on their phenolic compositions and geographical sources [35]. Statistical analysis was performed using MetaboAnalyst 5.0 software. Prior to the discriminatory analysis, the dataset was auto scaled and log transformed to correct for heteroscedasticity and over- and under-estimation of less abundant features [58]. Figure 3 shows the results of the PCA analysis. The ellipses drawn at the 95% confidence level account for 46% of the variation in the sample groups. As illustrated from the first two principal components, honeys that originated from Washington, Idaho, Colorado, and Utah were clearly separated while Texas honey was closely distributed around the origin of the score plot. An ultimate reason for such clustering can be caused by similar environmental conditions or identical phenolic profiles [61]. However, Idaho samples were influenced in the negative direction of PC1.

**Figure 4:**
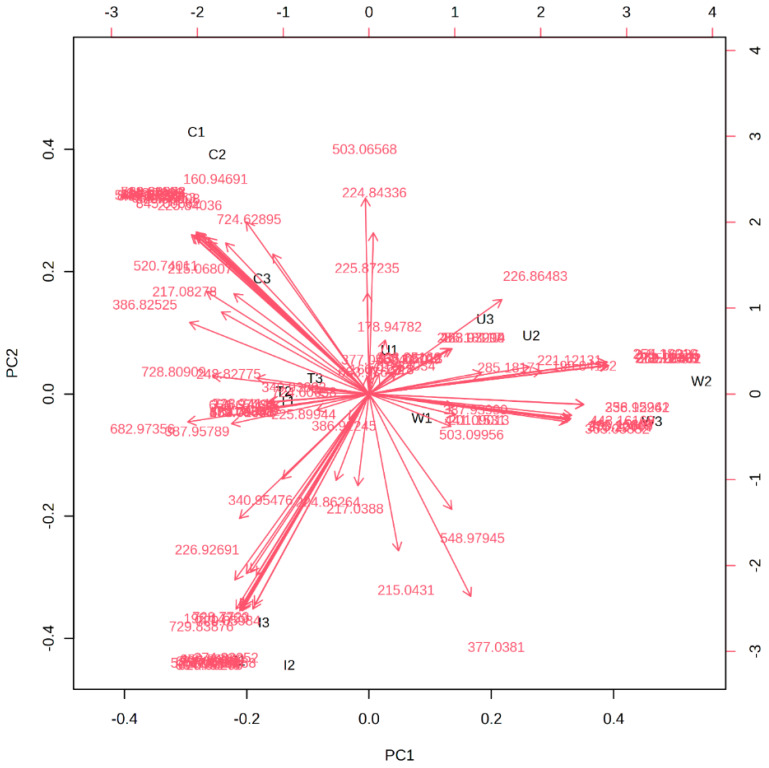
PCA biplot of the honey sample origins. The biplot shows the distribution of the sample features and their corresponding masses under the selected principal components.

**Figure 5:**
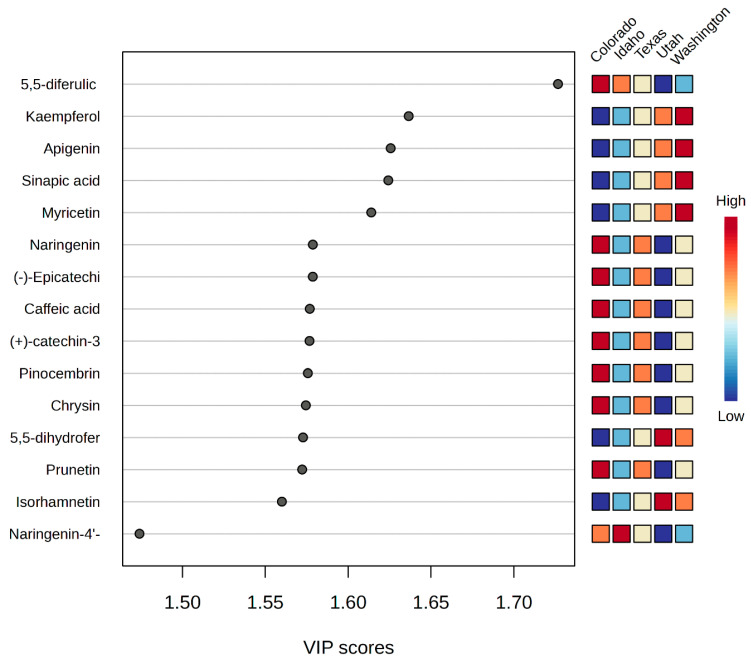
Important features identified by PLS-DA. The colored boxes on the right indicate the relative concentration of the corresponding compounds in each sample. The abbreviations for the left axis are as follows: (-)-epicatechin = ((-)-epicatechin-3-O-glucuronide; (+)-catechin = (+)-catechin-3-O-glucose; 5,5-dihydrofer = 5,5-dihydroferulic acid; and narigenin-4′- = naringenin-4′-glucoside.

Furthermore, PC1 was positively influenced by phenolic compounds in Washington honeys such as kaempferol, naringenin, apigenin, pinocembrin, and ferulic acid-5-5-dihydroferulic acid. Whereas sinapic acid and myricetin detected in Idaho honeys were responsible for its distribution in the negative region of PC1 in Figure 3. On the other hand, the positive trend observed for Colorado and Utah samples in PC2 can be explained by the presence of flavonoid glycosides: kaempferol-3-O-rhamnoside, (-)-epicatechin-4-O-glucuronide, 6-phenylnaringenin, and subaphyllin. Figure 4 is a PCA biplot of the honey sample origins. The clustering of Texas honey portrays a close overlap with the phenolic profiles of honeys from Colorado and Utah. The supervised PLS-DA technique was employed to extract the relevant features for the classification studies. Consequently, the variable importance in projection (VIP) scores from the PLS-DA model was used to extract the important variables for the discrimination of the honeys [62,63]. In this analysis, we performed cross validation and permutation tests to identify the best classifier and fitness of the data to the PLS-DA model. Based on the extracted VIP features in Figure 5, the phenolic compounds: 6-prenylnaringenin, kaempferol-3-O-rhamnoside, and subaphyllin, were tentatively identified as geographical markers for Utah honey. The flavonoid derivatives: catechin-3-O-glucose, caffeic-4-O-glucoside, and quercetin-3-O-(6″-malonyl-glucoside), and phenolic acids such as homovanillic acid and 5′5-dihydroferulic acid, were suggested as potentially relevant markers for both Colorado and Texas honeys. In the case of Washington and Idaho, kaempferol, apigenin pinocembrin, and myricetin were speculated as potential indicators of honeys from these regions. Moreover, our results show that caffeic acid can be suggested as a reliable marker for honey produced in Texas. Table 2 shows a detailed summary of the potential geographical markers found in the analyzed honeys. However, further studies may be crucial in authenticating the occurrence of the proposed markers in the investigated honeys. The distinct phenolic profiles and the occurrence of the compounds in the samples led to a more plausible classification of the honeys according to their geographical origins. Samples collected from Texas and Utah were grouped so closely that it was difficult to clearly differentiate them as displayed under the PCA plot in Figure 4. The honey samples from Colorado and Texas were saturated with high concentrations of the relevant features displayed in the VIP scale. Altogether, the PCA in combination with PLS-DA models are reliable tools to accurately classify the honeys according to their geographical regions.

## 3. Materials and Methods

### 3.1. Chemicals and Reagents

The Folin–Ciocalteu reagent and phenolic compound standards (chlorogenic acid, gentisic acid, sinapic acid, vanillic acid, gallic acid, p-coumaric acid, caffeic acid, apigenin, naringenin, rifampicin, quercetin, kaempferol) were obtained from Sigma Aldrich (St. Louis, MO, USA). HPLC grade methanol was acquired from Sigma Aldrich (St. Louis, MO, USA). Zinc acetate, sodium bisulfite, and sodium carbonate were purchased from Sigma Aldrich (St. Louis, MO, USA). Potassium ferrocyanide and hydrochloric acid were purchased from Fisher Scientific (Fair Lawn, NJ, USA). Ultrapure water was obtained from a Milli-Q water purification system (Nalgene, Rochester, NY, USA). Solid phase extraction (SPE) Sep-Pak C18 cartridges (500 mg, 3 mL) were purchased from Waters (Milford, MA, USA). Syringe filters (0.45 µm, diameter 25 mm) were acquired from Sterlitech (Auburn, WA, USA).

### 3.2. Collection of Honey Samples

Honey samples (n = 15) were directly received from a local hive honey company in air-tight sealed plastic containers. All honey samples were collected from beekeepers in different regions of the United States in January 2022. The samples originated from Texas, Colorado, Utah, Idaho, and Washington. There were no specific criteria or preferences in the selection of the sample origin. Information on the floral source of the honey was not available. The crystallized honeys were subjected to mild heating in a water bath and allowed to liquefy for 5 min. The samples were later stored at room temperature until further analysis.

### 3.3. Standards Preparation

A standard mix solution consisting of 7 phenolic acids (chlorogenic acid, gentisic acid, sinapic acid, vanillic acid, gallic acid, p-coumaric acid, and caffeic acid) and 5 flavonoids (apigenin, naringenin, rifampicin, quercetin, and kaempferol) was prepared at 1000 µg/L in methanol. Standard solutions of 100 µg/L and 50 µg/L were prepared by dilution of the stock mix solution. Standards were stored in the dark at 20 °C until further use. A calibration curve was generated by serial dilution of the working standard solution with a methanol-water mixture (20:80, *v*/*v*) at concentrations of 100, 50, 25, 12.5, 6.3, 3.3, 1.6, and 1.0 µg/mL.

### 3.4. Extraction of Phenolic Compounds in Honey

The honey samples were prepared according to the procedure described by Ciucure and Geană [19], with slight modifications. Briefly, a 10% (*w*/*v*) honey solution was prepared in 10 mL of acidified water (pH 2.0, 0.1 M HCl). The acidified honey solutions were homogenized using a magnetic stirrer for 15 min for complete dissolution of the honey, followed by centrifugation (5000× *g*, 30 min) to remove comb, propolis, and particulate matter. The SPE clean-up step was then carried out to remove sugars and other interfering matrix components. SPE was performed using the C18 cartridges. After conditioning with 5 mL of methanol and water, 2 mL of each prepared honey solution was passed through the C18 cartridge. The excess sugar residues were thoroughly washed with 5 mL of water followed by subsequent elution of the polyphenolic compounds with 2 mL of aqueous methanol. Following the extraction, the resulting phenolic extracts were filtered using a 0.45 µm syringe filter. Samples were aliquoted and stored at 25 °C room temperature prior to HPLC/MS analysis.

### 3.5. Determination of Total Polyphenol Content (TPC) in Honey

The total phenolic content in honey was determined with slight modifications in the method adapted from Singleton et al. [64]. The acidified honey solution (200 µL) was diluted with 5 mL of deionized water followed by addition of 100 µL Folin–Ciocalteu reagent. After vortexing, 300 µL of 20% sodium carbonate solution was added to the mixture. The samples were then homogenized and allowed to stand at room temperature in the dark for 2 h. Absorbance measurements were recorded in triplicate at a wavelength of 765 nm using a Cary 60 UV-Vis spectrophotometer (Agilent Technologies Inc., Santa Clara, CA, USA). The honey polyphenolic content was determined from a calibration curve made from gallic standards at the following concentrations (1, 2, 5, 10, 25, and 50 µg/mL). The TPC values are reported as gallic acid equivalents per gram of honey (mg GAE/g of honey, R^2^ = 0.9931).

### 3.6. Physicochemical Properties of Honey

#### 3.6.1. 5-Hydroxymethylfurfural (HMF) Determination in Honey

The HMF content in honey was tested according to White [62]. The method involves the use of clarified aqueous honey solutions against a reference standard of the same honey solution containing bisulfite. In this procedure, 5 g of honey was dissolved in deionized water (25 mL) and mixed with 0.5 mL of Carrez I solution (15% potassium ferrocyanide in DI water) and 0.5 mL of Carrez II solution (30% zinc acetate solution in DI water). After filtration, a 5 mL aliquot of the filtrate was added to two test tubes containing 5 mL of distilled water (sample) and 5 mL of 0.2% sodium bisulfite (reference), respectively. The absorbance of the sample against the reference was measured at 284 nm and 336 nm using a Cary 60 UV-Vis spectrophotometer. The HMF content in honey was determined using the proposed formula reported in the literature [65].

#### 3.6.2. Moisture Content in Honey

The moisture content in the honey samples was analyzed by using the detailed procedure given by Bogdanov [66]. A hand-held digital refractometer (MSC Industrial Inc., Shanghai, China) was initially calibrated with distilled water prior to the measurements. The surface of the refractometer was thoroughly cleaned and dried after each measurement to reduce spurious readings. Briefly, 0.1 g of the sample was placed directly on the surface of the prism and the reading was recorded after 1 min. All samples were analyzed in triplicate and the results reported as percent moisture content.

#### 3.6.3. pH Determination

To measure the pH of honey, a 10% honey solution was prepared in deionized water. The pH readings of the solutions were measured using a standardized pH meter.

#### 3.6.4. Color of Honey

The color of the honey was classified using the Pfund procedure and formula proposed by Beretta et al. [40]. More specifically, a 50% aqueous honey solution (*w*/*v*) was prepared and subjected to mild heating at 50 °C for complete dissolution of the sugar crystals. The measurements were carried out using a UV-Vis spectrophotometer at 635 nm. The absorbance readings were converted into mm Pfund values using Equation (1) [67],

(1) mm Pfund=−38.70+(371.39×Abs)

where Abs is the absorbance of the honey solution.

#### 3.6.5. Profiling of Polyphenols in Honey by HPLC-MS

All experiments were performed using a high-performance liquid chromatography system (Perkin Elmer, 200 Series, Waltham, MA, USA) coupled to a LCQ Deca XP+ ion trap mass spectrometer (Thermo Finnigan, San Jose, CA, USA). The separation of the phenolic compounds was achieved on a reversed phase discovery HS C18 column (3 µm × 2.1 mm × 150 mm, Millipore Sigma, Burlington, MA, USA) maintained at a temperature of 40 °C. A binary solvent system consisting of mobile phase A (water with 0.1% formic acid) and mobile phase B (methanol with 0.1% formic acid) was operated under the following gradient conditions: 0 min, 2%B; 0–5 min, 2%B; 5–15 min, 2–98%B; 15–20 min, 2–98%B; 20–30 min, 2%B for column equilibration run time. Furthermore, 10 µL of the honey solution was injected at a flow rate of 0.3 mL/min into the LC system for a total run time of 25 min. Honey phenolic profiling was based on the comparison of the retention times and experimental *m*/*z* values against the external standard analyzed under the same conditions. Mass spectrometry conditions were carried out with the ion trap mass spectrometer using electrospray ionization (ESI) operated in the negative ion mode. The ESI source was set using the following conditions: capillary temperature of 320 °C and an ionization voltage of 4.5 kV. MS spectra were acquired in the full scan mode within the *m*/*z* range of 100–1000 Da. The MS/MS fragmentation studies for each analyte were assisted using a data-dependent scan with nominal collision energies set between 20 and 50 eV and using He as the collision gas. The collision energies were optimized for each phenolic compound. LC-MS/MS data acquisition and processing were executed using Xcalibur 2.0.7 software (Thermo Finnigan, San Jose, CA, USA).

### 3.7. Statistical Analysis

One-way ANOVA was used to examine the differences between the phenolic composition and geographical origins of the honey samples according to the Fisher LSD method (*p* < 0.05). All samples were analyzed in triplicate and the results are expressed as the mean ± standard deviation (SD). The honey physicochemical properties were evaluated using the Pearson correlation coefficient. In addition, the principal component analysis (PCA) and partial least squares-discriminant analysis (PLS-DA) model were employed for honey classification. Data analysis was executed with MetaboAnalyst 5.0 software (MetaboAnalyst, McGill, CA, USA) and Minitab 21.1.1 software (Minitab ID: 2485). Briefly, one-way ANOVA was carried out in Minitab (Minitab ID: 2485) to compare the differences between the honey samples based on their geographical locations. The grouping information and test for differences of means was obtained using the Fisher LSD method at the 95% confidence level. The regression output obtained from Minitab was used to assess the effects of each measured physicochemical parameter on the TPC values of the honeys. The data analysis was also executed with MetaboAnalyst 5.0 by first grouping the peaks based on their *m*/*z* values, intensity, and retention time in a 3-column format. Peak matching across samples was compared by using the default mass tolerance (*m*/*z*) and retention time values of 0.025 and 30 s, respectively. Features that were identified with too many missing values were processed by applying the default settings suggested in MetaboAnalyst. Large variables and baseline noises were removed during data filtering using the default RSD threshold value of 20%. The data were subsequently normalized using log-transformation while sample normalization and data scaling parameters were set to “None”. Multivariate statistical analysis was performed using ANOVA, PCA, and PLS-DA in MetaboAnalyst. Under PCA, the dataset was further reduced into two dimensions (PC1 and PC2) which provided the greatest variation between the samples at the 95% confidence level. The combination of the biplot with PCA was particularly useful to demonstrate the relationship between the sample groups and identified mass features. To elucidate the separation between the groups, the supervised PLS-DA analysis was employed as both a classification and feature selection tool. The variable importance in projection (VIP) score was used to select the most significant features. A VIP cut-off value of 1.0 was selected for further data analysis. One-way ANOVA and post-hoc test were further implemented to compare the geographical origins of the honeys. The false discovery rate (FDR) and *p*-value threshold were set to the default values of 0.2 and 0.05, respectively.

## 4. Conclusions

In summary, this study provides insight into the phenolic composition of honeys produced in different regions in the United States. To the best of our knowledge, this study is the first to report the polyphenol content of honeys from selected geographical regions in the United States, which makes our findings relevant in the identification of characteristic markers in honeys from the different studied geographical sources. The polyphenol screening analysis detected the presence of 5 phenolic acids and 13 flavonoid compounds in all the samples, of which kaempferol, caffeic acid, 5′5-dihydroferulic acid, and 6-phenylnarigenin were identified as the main markers in honeys from Washington, Idaho, Texas, Colorado, and Utah, respectively. Our results indicated that the honeys can be classified into their geographical origins with the aid of chemometric tools such as PCA and PLS-DA. Moreover, the results of the total phenolic content of the studied samples revealed the highest abundance of phenolic compounds in Washington and Texas honeys. We observed a strong positive correlation between the total polyphenolic content and the color of the analyzed honeys. In contrast, the other examined parameters including pH, moisture content, and HMF did not significantly impact the phenolic content. Taken together, the individual fingerprinting of phenolic compounds in combination with chemometric models enabled the classification of honeys according to their geographical origins. A large number of highly diverse samples are crucial to establish an extensive database of markers for various honey authentication studies and this work is in progress.

## Figures and Tables

**Table 1:** Physicochemical properties of honey from different geographical origins.

Sample Origin	Color (mm Pfund Value)	%Water Content (g/100 g)	HMF Levels (mg/kg)	pH	TPC (mg GAE/100 g)
Colorado	Amber (88.8)	17.2	5.6	5.5	81.6
Idaho	Amber (102.8)	16.8	8.2	5.5	97.5
Texas	Dark amber (116.3)	18.2	9.9	5.4	100.2
Utah	Light amber (64.4)	16.4	5.5	5.4	84.3
Washington	Dark amber (118.2)	18.0	12.1	5.4	105.7
Codex Alimentarius Standard Commission (2001)	Nearly white to dark amber	<20%	<40 mg/kg or <80 mg/kg (regions with tropical temperatures)	3.5–6.0	
Regression coefficient (r)	0.931	0.036	0.467	0.169	

**Table 2:** Markers of geographical origin detected in honey.

Phenolic Compounds	Mass [M-H]^−^ (Da)	Makers of Geographical Origin	Polyphenol Class
Caffeic acid	178.95	Texas	Phenolic acid
Kaempferol	285.15	Washington	Flavonol
Apigenin	269.22	Washington	Flavone
Pinocembrin	255.18	Washington/Colorado	Flavanone
Subaphyllin	263.03	Utah	Phenolic acid
Kaempferol-3-O-rhamnoside	428.98	Utah	Flavanol glycoside
Ferulic acid-5-5-dihydroferulic acid	386.94	Colorado/Texas	Phenolic acid
6-Prenylnaringenin	338.85	Utah	Flavanone
(+)-Catechin 3-O-glucose	452.03	Colorado/Texas	Flavanol glycoside
Myricetin	316.82	Idaho	Flavanol
Quercetin 3-O-(6″-malonyl-glucoside)	548.96	Colorado	Flavonol
(-)-Epicatechin 4’-O-glucuronide	465.18	Utah	Flavonol

## Data Availability

The data presented in this study are available on request from the corresponding author.

## Supplementary Materials

The following supporting information can be downloaded at: https://www.mdpi.com/article/10.3390/molecules28135011/s1, Table S1: Characteristic retention times, exact mass, molecular mass, and fragment masses of identified phenolic compounds in honey.

## Sample Availability

Samples of the honey tested are not available from the authors.
