# Supplementary material for: Profiling of the Polyphenol Content of Honey from Different Geographical Origins in the United States
Source: Molecules. 2023 Jun 27;28(13):5011. doi: 10.3390/molecules28135011 (PMC10343218; doi:10.3390/molecules28135011)
Supplement: Supplementary file 1 [file molecules-28-05011-s001.zip › molecules-2437804-supplementary.pdf]

Profiling of the Polyphenol Content of Honey from Different Geographical Origins in the United States

Kate Nyarko [1], Kaitlyn Boozer [1] and C. Michael Greenlief [1,*]

[1] Department of Chemistry, University of Missouri, 601 S. College Avenue, Columbia, MO 65211, USA; knzhb@mail.missouri.edu (K.N.); kboozer@mail.missouri.edu (K.B.)

* Correspondence: greenliefm@missouri.edu; Tel.: +1-573-882-3288

Contents

Table S1: Characteristic retention times, exact mass, molecular mass, and fragment masses of identified phenolic compounds in honey.

Supplementary Table S1. Characteristic retention times, exact mass, molecular mass, and fragment masses of identified phenolic compounds in honey.

Compound	Retention time (min)	Molecular mass (Da)	Measured mass [M-H]$^-$, (Da)	Mass fragments (MS^2), 1st, (Da)	Mass fragments (MS^2), 2nd, (Da)	Collision energy (eV)	Sample origin
Phenolic acids							
Sinapic acid	1.32	224.0684	223.04	178.68	186.86	35	All origins
Caffeic acid	1.45	180.0422	178.96	150.87	134.85	35	All origins
5,5-dihydroferulic acid	1.50	386.100	385.12	340.65	236.87	35	All origins
Ferulic acid	1.18	194.0579	193.03	134.85	98.20	35	Utah
Flavonoids							
Myricetin	5.20	318.0375	316.63	151.73	179.12	40	Colorado/Texas/ Idaho
Quercetin 3-O-(6''-malonyl-glucoside)	12.49	550.0958	548.96	301.88	256.14	40	All origins
Isorhamnetin	7.38	316.0583	315.01	150.95	162.57	40	Washington
Quercetin	7.95	302.0426	301.10	201.32	153.27	40	Washington
(+)-Catechin 3-O-glucose	8.08	452.1318	451.02	199.68	254.12	40	Colorado/Texas
Kaempferol	8.70	286.0477	285.19	153.04	213.20	40	All origins
Naringenin	6.83	272.0684	271.13	148.89	153.23	40	Colorado/Utah/ Washington
Chrysin	11.60	254.0579	253.20	143.55	120.42	40	Colorado
Apigenin	9.54	270.0528	269.22	152.07	182.08	40	Washington
Pinocembrin	10.75	256.0735	255.21	153.22	171.62	40	Washington/ Colorado/Utah
Other flavonoids and phenolic acids							
6-phrenylnarigenin	10.09	340.1310	338.85	219.97	120.68	40	Utah
Hesperidin	11.05	610.1897	609.57	304.03	195.51	40	Idaho
Kaempferol 3-O-rhamnoside	15.03	431.0983	429.98	287.17	128.62	40	Utah
Subaphyllin	7.75	264.1473	263.03	179.25	152.11	40	Utah
